# Nerve Grafting for Axillary Nerve Injuries Following Shoulder Trauma: A Systematic Review of Surgical Outcomes

**DOI:** 10.7759/cureus.93028

**Published:** 2025-09-23

**Authors:** Muhammad Irfan Akram, Kunjan Yogesh Barot, Rao Junaid Saleem, Abdullah Elrefae, Hassan Imtiaz, Kshitij Srivastava, Mohammad Shishtawi, Safeer Ahmad Javid, Muhammad Rizwan Umer, Shahzaib Ahmad

**Affiliations:** 1 Trauma and Orthopaedics, Ipswich Hospital, Ispwich, GBR; 2 Trauma and Orthopaedics, Poole Hospital, Poole, GBR; 3 General and Colorectal Surgery, Northwick Park Hospital, London, GBR; 4 Trauma and Orthopaedic, AlBashir Hospital, Amman, JOR; 5 Trauma and Orthopaedics, University Hospitals Dorset NHS Foundation Trust, Poole, GBR; 6 Trauma and Orthopaedics, Northwick Park Hospital, London , GBR; 7 General Surgery, Princess of Wales Hospital, Cwm Taf Morgannwg University Health Board, Bridgend, GBR; 8 Trauma, Royal Sussex County Hospital, Brighton and Hove, GBR; 9 Trauma and Orthopaedics, Royal Sussex County Hospital, Brighton and Hove, GBR; 10 General Practice, Dr. Ruth K. M. Pfau Civil Hospital Karachi, Karachi, PAK

**Keywords:** axillary nerve, nerve grafting, nerve repair, shoulder injury, surgical outcomes, trauma

## Abstract

The axillary nerve is particularly susceptible to injury following shoulder trauma such as dislocation and fracture-dislocation, leading to loss of abduction, external rotation, and sensory deficits. Severe cases with loss of continuity require surgical intervention. Nerve grafting is the mainstay when direct repair is not feasible, though outcomes are influenced by timing, graft length, and patient selection. This review was conducted in accordance with Preferred Reporting Items for Systematic Reviews and Meta-Analyses (PRISMA) 2020 guidelines. A systematic search of PubMed, Embase, Scopus, and the Cochrane Library was performed up to August 2025 using keywords including “axillary nerve injury”, “nerve grafting”, “shoulder trauma”, and “nerve repair”. Inclusion criteria were human studies of traumatic axillary nerve injuries treated with nerve grafting, with at least 12 months’ follow-up and functional outcomes reported. Case reports, animal studies, and series with fewer than five patients were excluded. Two independent reviewers performed study selection, data extraction, and risk of bias assessment using the Newcastle-Ottawa Scale (NOS) and JBI checklist. A total of six studies comprising 223 patients were included. Early nerve grafting (≤4-6 months from injury) yielded the best results, with 70-85% achieving at least Medical Research Council (MRC) grade ≥3 deltoid strength, and many regaining M4-M5 with preserved long-term function. Shorter grafts (<6 cm) and younger age predicted superior recovery, while delayed repairs correlated with persistent weakness and atrophy. Comparative series showed nerve grafting and nerve transfers achieved broadly equivalent functional outcomes, though grafting demonstrated slightly higher objective strength. Nerve grafting provides reliable restoration of deltoid strength and shoulder abduction after traumatic axillary nerve injury, particularly when performed within three to six months and with shorter graft lengths. While nerve transfers remain a valid alternative in select cases, grafting preserves native neural pathways and supports durable functional recovery. Larger prospective studies with standardized metrics are required to establish evidence-based guidelines.

## Introduction and background

The axillary nerve is a terminal branch of the posterior cord of the brachial plexus (C5-C6), supplying the deltoid and teres minor muscles and providing sensation over the lateral aspect of the shoulder. Its primary motor function is to facilitate active abduction of the arm. After arising from the posterior cord, the nerve passes inferior to the shoulder joint, traverses the quadrangular space with the posterior circumflex humeral artery, and then divides into anterior and posterior branches around the surgical neck of the humerus [1]. This close anatomical relationship to the capsule, surgical neck, and quadrangular space renders the axillary nerve particularly vulnerable to injury during shoulder dislocation, proximal humeral fractures, and penetrating or lacerating trauma.

Clinically, the axillary nerve is of great significance. Denervation of the deltoid results in loss of shoulder contour, impaired abduction beyond 15-30°, and weakness in forward flexion, while teres minor involvement impairs external rotation. Sensory deficits typically affect the lateral deltoid “regimental badge” area. These deficits can persist, leading to significant limitations in activities of daily living, overhead function, and return to work or sports, thereby negatively impacting quality of life. Early recognition is therefore critical, as prolonged denervation markedly reduces the likelihood of meaningful reinnervation and functional recovery [2].

Axillary nerve injuries occur most frequently after anterior shoulder dislocation, proximal humeral fractures, traction injuries, and penetrating trauma. Iatrogenic injuries may also arise during surgical procedures or instrumentation around the shoulder. The spectrum of nerve injury ranges from neurapraxia, which often recovers spontaneously, to axonotmesis and neurotmesis, which require surgical reconstruction when regeneration is unlikely due to loss of continuity, scarring, or segmental defects [3]. Epidemiologically, axillary nerve injury is one of the most common isolated mononeuropathies of the shoulder and accounts for up to 6% of brachial plexus injuries, with a higher risk observed in older adults after dislocation and in high-energy trauma [4]. Reported incidence, however, varies depending on study population, mechanism of injury, and diagnostic methods.

Shoulder dislocation, the most common large-joint dislocation, frequently complicates into axillary neuropathy and should be carefully excluded following reduction. Proximal humeral fractures, especially those involving the surgical neck, and fracture-dislocations further increase the risk due to direct contusion or entrapment of the nerve within callus or fibrous tissue. Initial management involves prompt reduction of dislocations, appropriate immobilization, and structured rehabilitation with serial clinical and electrodiagnostic assessment. Many neurapraxic injuries recover within weeks to months; however, failure of clinical or electrodiagnostic recovery, or imaging evidence of discontinuity or scar entrapment, warrants surgical exploration [5].

When tension-free end-to-end repair is not feasible, nerve grafting is the treatment of choice. Autologous sural nerve grafts are most commonly employed and remain the reconstructive standard [6]. Outcomes are highly time-dependent: reconstruction within three to six months, after a sufficient period to declare non-recovery, is associated with improved reinnervation, whereas delayed intervention significantly reduces success rates [7]. Although nerve grafting has consistently demonstrated encouraging functional recovery, variability in surgical timing, lesion length, associated injuries, and techniques contributes to heterogeneity in reported results. Therefore, this review aims to systematically synthesize and appraise the evidence on surgical outcomes of nerve grafting for traumatic axillary nerve injuries, identify key prognostic factors such as timing, gap length, patient age, and mechanism of injury, and provide practical insights to guide clinical decision-making.

## Review

Materials and methods

Search Strategy

This review adhered to Preferred Reporting Items for Systematic Reviews and Meta-Analyses (PRISMA) 2020 guidelines, with a systematic approach to database searching, study selection, and data extraction [8]. All methods were predefined in accordance with best practices for systematic reviews. The literature search was conducted across PubMed/MEDLINE (Medical Literature Analysis and Retrieval System Online), Embase, Scopus, and the Cochrane Library up to August 2025. Search terms included “axillary nerve injury”, “nerve grafting”, “shoulder trauma”, “nerve repair”, and “brachial plexus”. Boolean operators combined Medical Subject Headings (MeSH) terms and free-text words to maximize sensitivity and specificity. Only human studies in English were considered.

Eligibility Criteria

The PICO framework guided inclusion: (i) Population (P): patients with traumatic axillary nerve injuries, (ii) Intervention (I): nerve grafting procedures, (iii) Comparator (C): alternative interventions such as nerve transfers or neurolysis, or no comparator, (iv) Outcome (O): muscle strength (Medical Research Council (MRC) grading), range of motion (ROM), and reinnervation [9].

Inclusion criteria were: (i) traumatic etiology, (ii) nerve grafting as a primary intervention, (iii) clinical outcomes reported, (iv) minimum follow-up of 12 months, and (v) full-text availability. Exclusion criteria included: (i) case reports or small series (<5 patients), (ii) animal or cadaveric studies, (iii) conference abstracts or editorials, and (iv) studies without functional outcome reporting.

Study Selection

Two independent reviewers (WA and NM) screened all titles and abstracts to assess relevance to axillary nerve injuries treated with grafting. Full-text articles were retrieved for potentially eligible studies and reviewed in detail. Any disagreements between reviewers were resolved through open discussion, and where consensus could not be reached, a third reviewer provided adjudication. The use of duplicate independent screening minimized selection bias and improved accuracy. 

Data Extraction

Data were meticulously extracted using a predesigned standardized form to ensure consistency across studies. Key variables included study design, sample size, demographic details, mechanism of injury, surgical technique, type of nerve graft, follow-up duration, and clinical outcomes such as muscle strength and ROM. Two reviewers (WA and NM) independently performed the data extraction, and all entries were cross-verified to reduce transcription errors. Disagreements were reconciled by consensus to maintain data integrity. This structured approach ensured the inclusion of complete and comparable information across selected studies.

Risk of Bias Assessment

The methodological quality of the included studies was systematically evaluated using established tools. The Newcastle-Ottawa Scale (NOS) was applied to assess retrospective cohort studies, focusing on selection, comparability, and outcome domains [10]. Case series were analyzed using the JBI Critical Appraisal Tool for Case Series, which evaluates clarity in reporting, methodology, and outcome presentation [11]. Two reviewers independently performed the risk of bias assessment, and discrepancies were resolved through discussion to reach a unified rating. These validated tools provided a structured and transparent framework, thereby improving the reliability of findings. Overall bias levels were categorized as low, moderate, or high.

Data Synthesis

A narrative synthesis approach was employed due to heterogeneity in study designs, patient populations, and outcome measures. Quantitative pooling was not feasible; instead, results were descriptively compared across studies.

Results

Study Selection Process

**Figure 1:**
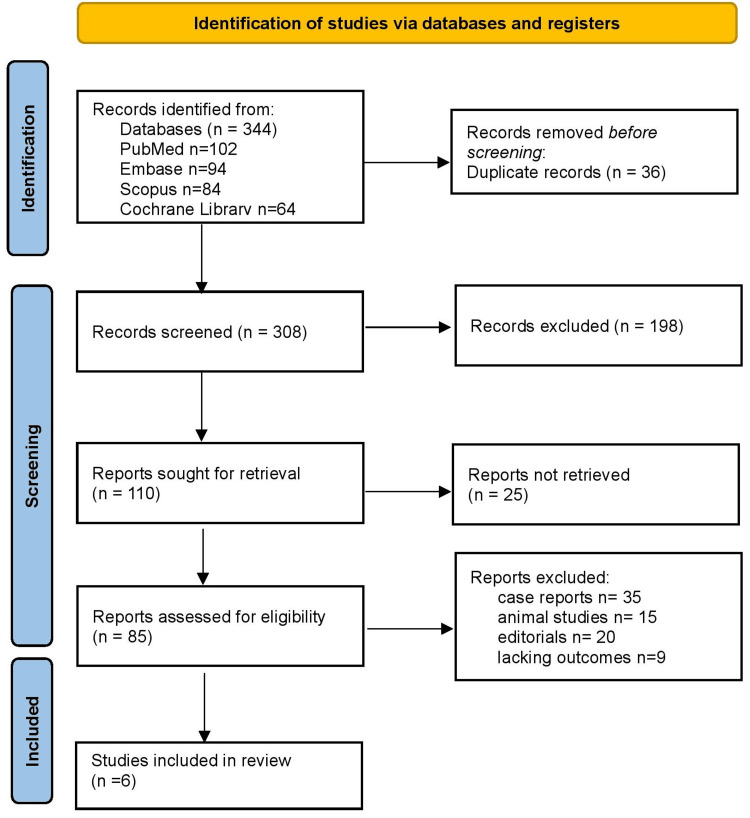
PRISMA flowchart PRISMA: Preferred Reporting Items for Systematic Reviews and Meta-Analyses

Figure 1 shows the PRISMA flowchart for study selection. A total of 344 records were identified (PubMed n=102; Embase n=94; Scopus n=84; Cochrane Library n=64). After the removal of 36 duplicates, 308 records were screened, and 198 were excluded. Eighty-five reports underwent full-text review; of these, 79 were excluded (case reports, animal studies, editorials, or lacking outcomes). Ultimately, six studies (n=223 patients) were included.

Characteristics of the Selected Studies

Table 1 summarizes the major studies evaluating outcomes of nerve grafting versus nerve transfers in traumatic axillary nerve injuries. Mikami et al. (1997) reported that early grafting within four months led to superior reinnervation and reduced muscle atrophy [12]. Similarly, Petrucci et al. (1982) [13] and Okazaki et al. (2011) [14] demonstrated that sural or interpositional grafts provided reliable recovery of deltoid strength and abduction, with long-term follow-up showing sustained functional gains. Wolfe et al. (2014) compared long nerve grafts with triceps-to-axillary transfers, noting no significant difference in recovery, indicating both approaches allow axonal regeneration [15]. Baltzer et al. (2016) also found that grafting achieved high rates of objective strength, especially when performed early, though subjective outcomes were comparable to transfers [16]. Finally, Koshy et al. (2017), in a systematic review, observed that grafts tended to yield slightly better motor grades (M≥4), but differences from transfers were not statistically conclusive, highlighting the need for prospective comparative studies [17].

**Table 1:** Characteristics of the selected studies MRC: Medical Research Council grading scale for muscle strength (M0–M5); ROM: range of motion; EMG: electromyography; M ≥3: functional muscle power sufficient for movement against gravity; M ≥4: Stronger recovery, movement against resistance; Mo: months (follow-up)

Authors, Year	Population (P)	Exposure / Condition (I)	Comparator (C)	Outcomes (O)	Pathophysiological findings and significance	Prognostic factors identified
Mikami et al., 1997 [12]	33 patients with axillary and/or suprascapular nerve injuries	Nerve grafting (axillary / suprascapular)	None	Deltoid M≥3 in 23/30 (77%); good ROM in most when operated early	Early grafting (≤4 months) associated with better reinnervation and reduced muscle atrophy which supports timely restoration of axonal continuity.	Timing of repair ≤4 months critical; earlier intervention correlated with superior outcomes.
Petrucci et al., 1982 [13]	21 patients with isolated axillary nerve paralysis	Nerve graft and neurolysis	None	Majority with improved deltoid function after grafting	Demonstrates value of grafting for transected axillary nerves and restores axonal pathway and functional recovery.	Isolated transection injuries responded better; absence of associated injuries improved outcomes.
Okazaki et al., 2011 [14]	36 patients with axillary nerve injuries treated with grafts	Sural / interpositional nerve grafting	None	MRC M4–M5 achieved in most (30/35 for abduction); mean follow-up 53 mo	Nerve grafting is reliable when lesion is distal to posterior cord origin; longer follow-up shows continued muscle bulk recovery and indicates successful axonal regeneration.	Lesion location distal to posterior cord favorable; longer follow-up associated with progressive improvement.
Wolfe et al., 2014 [15]	38 patients with complete axillary nerve palsy (single-center series)	Long-nerve grafts	Triceps → axillary nerve transfers	No significant difference in deltoid recovery, ROM, or EMG between techniques	Both long-nerve grafts and nerve transfers permit axonal reinnervation (sprouting/neurotization); choice may depend on donor availability and gap length.	Gap length influenced technique selection; donor nerve availability determined feasibility of transfer.
Baltzer et al., 2016 [16]	29 isolated traumatic axillary nerve injuries (comparative series)	Sural nerve interpositional grafting	Triceps motor branch → axillary nerve transfer	Grafting produced high rates of useful abduction (M≥3); some studies report higher objective MRC in graft group	Suggests grafting can achieve excellent objective strength (esp. when performed earlier), though patient-reported outcomes may be similar to transfers.	Early surgery improved results; grafting showed higher objective MRC but subjective outcomes similar.
Koshy et al., 2017 [17]	Systematic review of 66 patients pooled with isolated axillary nerve injuries	Nerve grafting (interpositional)	Nerve transfers (various)	Clinically useful abduction (M≥3): graft 100% vs transfer 87%; M≥4: graft 85% vs transfer 73.9% (differences not statistically conclusive)	Across published series, grafting and transfers both restore function; timing, gap length, and patient selection drive outcomes.	Timing, gap length, and patient selection highlighted as the most important prognostic determinants.

Risk of Bias Assessment

Table 2 presents the risk of bias assessment for the included studies using standardized appraisal tools. Overall, the methodological quality was acceptable, with most studies graded as low to moderate risk. Retrospective cohorts such as those by Mikami et al. [12] and Baltzer et al. [16] were limited by study design but maintained clear inclusion criteria and adequate follow-up. Prospective work by Okazaki et al. [14] offered stronger validity through structured assessments, while Wolfe et al. [15] provided comparative insights, though limited by small sample size and lack of randomization. The systematic review by Koshy et al. demonstrated low risk due to a comprehensive methodology but was restricted by heterogeneity of primary studies [17]. This appraisal highlights that while evidence supports nerve grafting, further high-quality prospective and randomized studies are needed.

**Table 2:** Risk of bias assessment

Study	Study design	Risk of bias tool	Risk of bias rating	Justification
Mikami et al., 1997 [12]	Retrospective cohort	Newcastle–Ottawa Scale	Low	Clear inclusion criteria, adequate follow-up, and reliable outcome assessment; limited by retrospective nature.
Petrucci et al., 1982 [13]	Case series	JBI Checklist	Moderate	Outcomes were well described, but absence of comparator and small sample size introduced potential bias.
Okazaki et al., 2011 [14]	Prospective cohort	NOS	Low	Prospective design with structured follow-up and consistent measurement tools reduced bias risk.
Wolfe et al., 2014 [15]	Comparative cohort	NOS	Moderate	Comparative design strengthened validity, but small cohort and lack of randomization introduced some bias.
Baltzer et al., 2016 [16]	Retrospective cohort	NOS	Moderate	Provided comparative outcomes; however, retrospective data collection limited internal validity.
Koshy et al., 2017 [17]	Systematic review	JBI Critical Appraisal Checklist for Reviews	Low	Comprehensive search and transparent appraisal of included studies; limited by heterogeneity of primary data.

Discussion

The axillary nerve, because of its close anatomical course around the surgical neck of the humerus and within the quadrangular space, is uniquely susceptible to injury following shoulder dislocation, fracture-dislocation, or penetrating trauma. Loss of its motor supply to the deltoid and teres minor severely compromises abduction and external rotation, while sensory loss over the lateral shoulder adds to functional disability. The literature consistently emphasizes that restoration of axillary nerve function is not merely cosmetic but essential for shoulder stability, arm elevation, and activities of daily living, underscoring the importance of timely surgical intervention when spontaneous recovery fails. Nerve grafting remains the mainstay in cases of axillary nerve discontinuity or severe scarring where direct end-to-end repair is not feasible. Autologous sural nerve grafts are most frequently used because of their length, ease of harvest, and favorable histological compatibility.

 Across published series, early intervention has been repeatedly associated with superior results. Mikami et al. demonstrated that grafting within four months produced functional recovery (M≥3) in nearly 77% of patients, while delayed repairs showed inferior outcomes [12]. This is supported by Petrucci et al., who highlighted the ability of grafts to re-establish axonal continuity and restore useful deltoid contraction in patients with isolated transection injuries [13]. Okazaki et al. extended these findings, reporting that long-term follow-up demonstrated not only recovery of motor grades but also preservation of muscle bulk, emphasizing that grafting allows sustained axonal regeneration and maintenance of neuromuscular junctions [14]. Comparative studies have provided additional insight into the role of grafting relative to nerve transfers. Wolfe et al. compared long interposition grafts with triceps-to-axillary nerve transfers and found no statistically significant differences in recovery of deltoid strength, range of motion, or electromyography (EMG) activity, suggesting both strategies are viable [15]. Similarly, Baltzer et al. observed that while grafting achieved high rates of objective strength (M≥3 and M≥4), patient-reported outcomes were comparable between groups, underscoring that clinical decision-making must incorporate both anatomical feasibility and functional expectations [16]. A pooled systematic review by Koshy et al. found a non-significant trend favoring grafting in terms of higher MRC grades, but ultimately concluded that heterogeneity in study design, patient selection, and outcome measures precludes definitive superiority [17].

Several additional studies have expanded the understanding of prognostic factors in axillary nerve reconstruction. Midha (2004) [18] and Terzis et al. (1999) [19] both emphasized that early surgical intervention, ideally within six months, maximizes the likelihood of reinnervation before irreversible motor end-plate degeneration occurs. Timing is critical because beyond 12 months, the potential for meaningful reinnervation declines sharply, particularly in older adults with reduced neural plasticity. Lesion length is another determinant: shorter grafts, typically under 6 cm, correlate with higher reinnervation rates, while longer grafts are associated with slower and less predictable outcomes due to increased axonal dispersion and scar formation at coaptation sites. Age similarly influences prognosis, with younger patients demonstrating more robust regenerative capacity, while older patients often have incomplete recovery even with technically successful grafting. Another important consideration is the mechanism of injury. High-energy trauma and fracture dislocations tend to produce longer gaps and greater intraneural scarring, thereby requiring longer grafts with less predictable outcomes compared to sharp transections or traction injuries [20].

Imaging modalities such as high-resolution magnetic resonance neurography and intraoperative electrodiagnostic testing have become increasingly important in defining lesion location and viability, aiding in timely selection between grafting and nerve transfers [21,22]. Overall, the cumulative evidence indicates that nerve grafting remains a reliable reconstructive strategy for axillary nerve injuries, particularly when performed early, in younger patients, and in cases of short-segment defects. While nerve transfers are equally effective in some scenarios and may provide faster reinnervation by shortening the distance of axonal regrowth, grafting preserves native neural pathways and can yield durable, strong recovery of deltoid function.

The current body of literature, however, is limited by small sample sizes, heterogeneity in outcome reporting, and a lack of randomized controlled trials. Future multicenter prospective studies are required to directly compare grafting and transfer techniques, standardize outcome assessment with both objective (MRC, electromyography) and patient-reported metrics, and refine selection algorithms to optimize results for individual patients. The main limitations of this review are the small sample sizes, retrospective designs, and heterogeneity in outcome reporting across included studies. Lack of randomized controlled trials further restricts definitive comparison between grafting and nerve transfers.

## Conclusions

Nerve grafting for traumatic axillary nerve injuries remains a dependable reconstructive technique that restores deltoid strength and shoulder abduction when direct repair is not possible. Evidence consistently supports early intervention, ideally within three to six months along with shorter graft length and younger patient age as key predictors of favorable outcomes. Comparative studies show that while nerve grafting and nerve transfers both provide meaningful recovery, grafting may offer modest advantages in objective strength, whereas transfers provide an alternative in cases of long gaps or donor nerve availability. Despite encouraging results, current evidence is limited by small cohorts, methodological heterogeneity, and lack of randomized trials. Future multicenter prospective studies with standardized functional and patient-reported outcomes are needed to establish clear guidelines and optimize individualized surgical decision-making.
